# Long-Term Efficacy of Catheter Ablation for Atrial Fibrillation in Different Phenotypes of Hypertrophic Cardiomyopathy

**DOI:** 10.31083/RCM41914

**Published:** 2025-10-29

**Authors:** Wei Du, Xinguang Chen, Yan Dong, Qiushi Chen, Nishant Yadav, Fengxiang Zhang

**Affiliations:** ^1^Division of Cardiology, The First Affiliated Hospital of Nanjing Medical University, 210029 Nanjing, Jiangsu, China; ^2^Division of Cardiology, Xuzhou Central Hospital, 221009 Xuzhou, Jiangsu, China; ^3^Section of Pacing and Electrophysiology, Division of Cardiology, The First Affiliated Hospital of Gannan Medical University, 341000 Ganzhou, Jiangxi, China

**Keywords:** apical hypertrophic cardiomyopathy, atrial fibrillation, catheter ablation, septal hypertrophic cardiomyopathy, recurrence

## Abstract

**Background:**

Atrial fibrillation catheter ablation (AFCA) success rates vary across different phenotypes of hypertrophic cardiomyopathy (HCM). Therefore, we compared long-term outcomes between apical (aHCM) and septal (sHCM) subtypes of HCM.

**Methods:**

This retrospective study analyzed patients with HCM who underwent AFCA at the First Affiliated Hospital of Nanjing Medical University between January 2010 and December 2020.

**Results:**

A total of 36 patients with aHCM and 80 patients with sHCM were enrolled. During a median follow-up of 42 months (interquartile range (IQR) 18–83), the overall atrial tachyarrhythmia (ATa) recurrence rate after a single ablation was 42.2% (49/116). The aHCM patients had a higher ATa recurrence rate than the sHCM patients (58.3% vs. 35.0%; χ^2^ = 5.54; *p* = 0.019). The ATa recurrence risk increased by 94% in patients with aHCM (hazard ratio (HR) 1.94, 95% confidence interval (CI) 1.10–3.43; log-rank *p* = 0.021). Subgroup analysis demonstrated pronounced risk elevation in paroxysmal atrial fibrillation (AF) patients (HR 2.85, 95% CI 1.44–5.67; *p* = 0.003), while no intergroup difference was observed in patients with persistent AF (HR 0.90, 95% CI 0.31–2.62; *p* = 0.853) (interaction *p* = 0.080). Multivariate Cox regression analysis identified antiarrhythmic drug (AAD) use (HR 0.22, 95% CI 0.08–0.59; *p* = 0.003), hypertension comorbidity (HR 2.50, 95% CI 1.21–5.19; *p* = 0.014), persistent AF type (HR 0.41, 95% CI 0.17–1.00; *p* = 0.049), and left atrial diameter ≥45 mm (HR 2.55, 95% CI 1.11–5.85; *p* = 0.028) as independent predictors of postoperative recurrence.

**Conclusions:**

An aHCM subtype predicts higher ATa recurrence after a single ablation versus sHCM. Hypertension, a left atrial enlargement ≥45 mm, and no AAD use are independent predictors of recurrence. Meanwhile, optimizing blood pressure and AAD therapy may improve outcomes.

## 1. Introduction

Atrial fibrillation (AF) affects 22.5% of hypertrophic cardiomyopathy (HCM) patients [1, 2]. It increases heart failure risk and mortality [3, 4]. Drug treatment is limited to maintaining sinus rhythm (SR) yet may cause serious adverse effects [5]. Catheter ablation (CA) offers an alternative approach. Prior studies [6, 7] support the feasibility and relative safety of CA for managing AF in HCM. However, success rates differ, and there is a frequent need for repeat procedures [8]. Depending on the predominant localization of segmental myocardial hypertrophy, HCM has distinct subtypes: septal (sHCM) and apical (aHCM) [9]. The potential differential effects of CA for AF among HCM phenotypes are not well established. Given the limited and inconsistent evidence, we directly compared ablation outcomes between these phenotypes.

## 2. Materials and Methods

### 2.1 Patient Population

Patients with HCM and AF who underwent *de novo* CA at the First Affiliated Hospital of Nanjing Medical University between January 2010 and December 2020 were included in this retrospective study. Exclusion criteria: (1) Individuals with prior CA for AF; (2) Patients presenting with severe comorbidities; (3) Cases with incomplete peri-procedural documentation; (4) Age <18 years or >80 years at enrollment; (5) History of structural cardiac interventions (alcohol septal ablation, surgical myectomy, or percutaneous transluminal septal myocardial ablation); (6) Scheduled for concomitant cardiac surgery; and (7) those lost to follow-up after the procedure.

All patients received effective pre-procedural anticoagulation. Transesophageal echocardiography or computerized tomography (CT) excluded cardiac thrombi. This study was conducted by following the Declaration of Helsinki and was approved by the Ethics Committee of the First Affiliated Hospital of Nanjing Medical University (ethics approval number: 2020-SR-494). Informed consent was waived due to the retrospective nature of the study.

### 2.2 Definitions

AF was categorized as either paroxysmal or persistent based on the established guidelines [10]. Paroxysmal AF is defined as a continuous episode lasting longer than 30 seconds but resolving spontaneously or through intervention within seven days. In contrast, persistent AF lasts longer than seven days but less than one year in duration. The diagnosis of HCM was made according to the 2011 American Heart Association (AHA) guidelines [11], which require that the left ventricular (LV) end-diastolic wall thickness be ≥15 mm in any segment as assessed by echocardiography or cardiac magnetic resonance imaging (CMRI). This diagnosis also requires the exclusion of other conditions, such as valvular heart disease or hypertension, that may cause similar degrees of left ventricular hypertrophy. The diagnostic criteria for aHCM [12] included demonstration of asymmetric LV hypertrophy, confined predominantly to the LV apex, with an apical wall thickness ≥15 mm and a ratio of maximal apical to posterior wall thickness ≥1.5. For sHCM, study enrollment was limited to patients exhibiting reverse-curve septal hypertrophy whenever feasible. Obstructive HCM (HOCM): Resting or provoked left ventricular outflow tract gradient (LVOTG) ≥30 mmHg [11].

### 2.3 The Ablation Procedure

All non-amiodarone antiarrhythmic medications were discontinued more than five half-lives pre-procedure. Local anesthesia was used during the procedures. Systemic anticoagulation was achieved through intravenous administration of heparin, maintaining an activated clotting time of 300–350 seconds throughout the procedure. The standardized Atrial fibrillation catheter ablation (AFCA) protocol used at our institution has been described in previous studies [13, 14]. A three-dimensional electroanatomical mapping system (CARTO, Biosense Webster) guided the ablation procedure. Complete circumferential pulmonary veins isolation (CPVI) was achieved using an Ablation index (AI)-guided approach, producing continuous circular lesions at a power of 30 to 40 W and contact force of 15 ± 5 g for all patients. The target AI values were set at 500 for anterior, 400 to 450 for roof, and 350 to 400 for inferior and posterior segments. Concomitant atrial flutter (AFL) or atrial tachycardias (AT) were ablated during the procedure. If AF persisted after CPVI, SR was restored by electrical cardioversion. Voltage mapping was performed during SR to identify the low voltage zones (voltage range: 0.1–0.4 mV) and transitional zones (voltage range: 0.4–1.3 mV). Homogenization of the low-voltage zones and elimination of the complex electrograms from the transitional zones were carried out. Superior vena cava isolation and cavotricuspid isthmus isolation ablation were performed if indicated.

### 2.4 Post-Ablation Management and Follow-up

Anticoagulation was recommended for a minimum of three months following ablation in patients with paroxysmal AF and for at least six months in those with persistent AF. Antiarrhythmic drugs (AADs) were resumed but then stopped after a 3-month post-ablation blanking period. Patients with blanking recurrence were treated with AADs and/or cardioversion if needed. All patients underwent scheduled follow-up assessments at our outpatient clinics at 1, 3, 6, 12 months postoperatively, as well as 6 months thereafter. A recurrence of atrial tachyarrhythmia (ATa) was defined as the return of AF/AFL/AT lasting more than 30 s on the standard Electrocardiograph (ECG), or 24-h Holter recording during the follow-up period after the 3-month post-ablation blanking period.

### 2.5 Statistical Analysis

Normally distributed continuous variables were expressed as mean ± standard deviation (SD) and compared using the Student *t*-test; Non-normally distributed data were presented as the median with interquartile range [IQR: Q1, Q3] and compared by the Mann-Whitney U test. Categorical variables were presented as frequency (percentage) and compared by χ^2^ tests or Fisher’s exact tests. Time to ATa recurrence was calculated using the Kaplan–Meier analysis and compared by log-rank statistics. Univariate and multivariate Cox analysis were used to test predictors for recurrence. Variables with a *p-*value < 0.1 in univariate analyses were included in stepwise multivariate Cox regression models. Statistical significance was defined as a two-tailed *p*-value < 0.05.

## 3. Results

### 3.1 Study Population

**Fig. 1.**
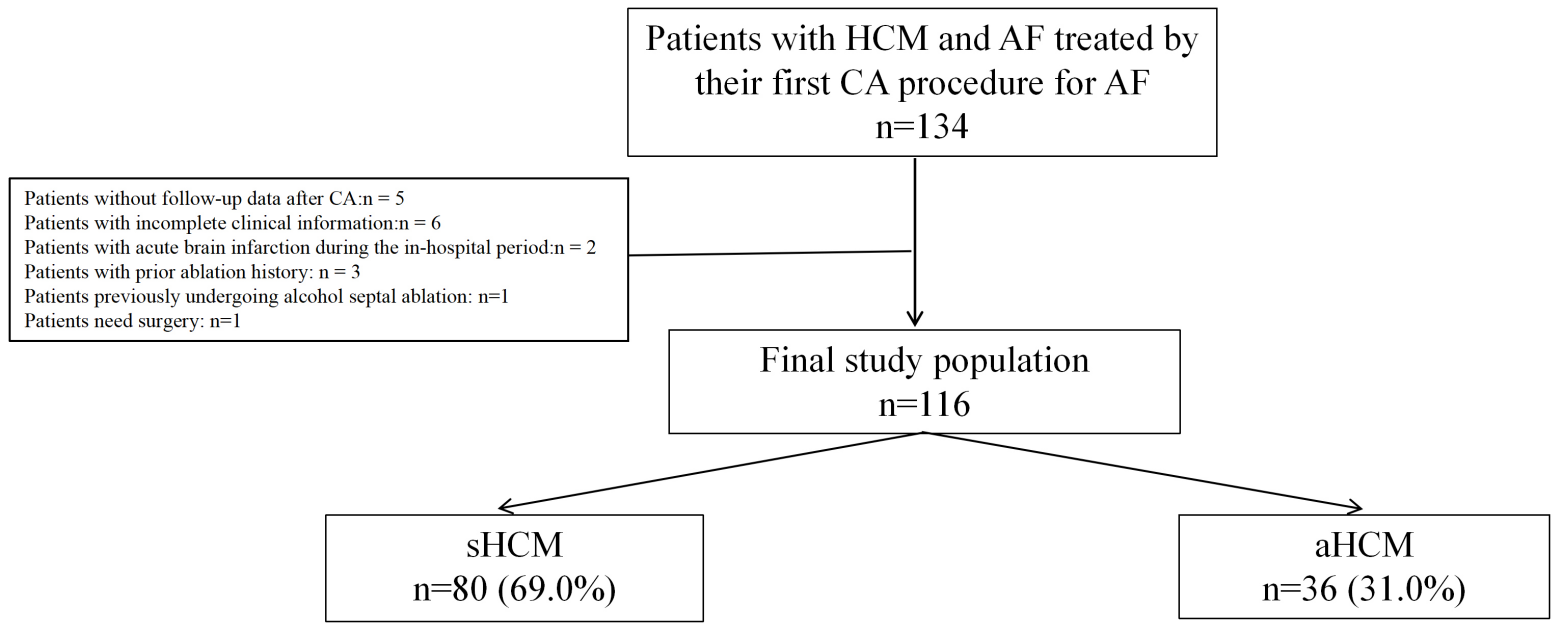
**Flow diagram for this study**. HCM, hypertrophic cardiomyopathy; AF, atrial fibrillation; CA, catheter ablation; sHCM, septal hypertrophic cardiomyopathy; aHCM, apical hypertrophic cardiomyopathy.

Between January 2010 and December 2020, 134 consecutive patients with HCM and drug-refractory symptomatic AF undergoing first CA were enrolled. After excluding five cases who failed to complete follow-up, six with incomplete clinical data, one who had previously undergone alcohol septal ablation, one who required surgery, three with prior history of ablation, and two who experienced acute brain infarction during hospitalization, a total of 116 individuals were included in the study: 36 aHCM, 80 sHCM (Fig. 1). The mean age was 57.9 ± 11.2 years; 82 (70.7%) were male. Baseline characteristics are displayed in Table 1. aHCMs were older and had more hypertension. AF type, AF duration, and drug usage (β-blockers and oral anticoagulants) were similar between groups. The mean septal thickness in the overall cohort was 16.1 ± 3.6 mm. Among the subgroup of 9 patients diagnosed with HOCM, the mean resting LVOTG was 31.4 ± 13.9 mmHg. Notably, 33.3% of these patients demonstrated provoked LVOT gradients of ≥30 mmHg. Typical systolic anterior motion of the mitral valve occurred in 3 of 9 HOCM patients.

**Table 1.** **Baseline characteristics**.

Variables	All patients	sHCM	aHCM	*p*-value
	(n = 116)	(n = 80)	(n = 36)	
Age (yrs)	57.9 ± 11.2	56.3 ± 11.8	61.4 ± 9.1	0.024
Male, n (%)	82 (70.7)	58 (72.5)	24 (66.7)	0.523
Height (cm)	170.5 ± 7.8	170.8 ± 7.8	170.0 ± 8.0	0.645
Weight (kg)	70.6 ± 10.6	70.7 ± 10.8	70.3 ± 10.2	0.814
Persistent AF, n (%)	34 (29.3)	23 (28.7)	11 (30.6)	0.843
AF duration (month)	36 (12.0, 72.0)	36 (12.0, 72.8)	48 (25.5, 72.0)	0.596
Hypertension, n (%)	54 (46.6)	32 (40.0)	22 (61.1)	0.035
Diabetes, n (%)	21 (18.1)	17 (21.3)	4 (11.1)	0.190
CHA_2_DS_2_-VASC	1.7 ± 1.4	1.5 ± 1.4	2.0 ± 1.4	0.122
LAD (mm)	45.3 ± 5.5	45.1 ± 5.8	45.8 ± 4.7	0.485
RAD (mm)	36.9 ± 4.7	36.9± 4.8	36.8 ± 4.5	0.941
LVEDD (mm)	47.1 ± 4.1	46.8 ± 4.3	47.9 ± 3.3	0.117
LVEDS (mm)	30.6 ± 4.3	30.5 ± 5.0	30.8 ± 2.1	0.643
LVEF (%)	63.5 ± 4.1	63.0 ± 4.5	64.7 ± 2.9	0.037
IVS (mm)	16.1 ± 3.6	16.5 ± 3.5	12.9 ± 1.1	<0.001
LVOTO (%)	9 (7.8)	9 (11.3)	-	NA
LVOTG (mmHg)	-	31.4 ± 13.9	-	NA
LVPW (mm)	11.2 ± 3.8	11.5 ± 4.3	10.7 ± 2.3	0.304
AADs, n (%)	109 (94.0)	75 (93.8)	34 (94.4)	1.000*
β receptor blocker, n (%)	90 (77.6)	66 (82.5)	24 (66.7)	0.059
ACEI/ARB, n (%)	40 (34.5)	32 (40.0)	8 (22.2)	0.062
Calcium antagonist, n (%)	17 (14.7)	8 (10.0)	9 (25.0)	0.035
NOACs, n (%)	39 (33.6)	25 (31.3)	14 (38.9)	0.420
Warfarin, n (%)	69 (59.5)	47 (58.8)	22 (61.1)	0.811

ACEI/ARB, angiotensin-converting enzyme inhibitor/angiotensin II receptor blocker; AF, atrial fibrillation; NOACs, non-vitamin K antagonist oral anticoagulants; LAD, left atrial diameter; RAD, right atrial diameter; LVEDD, left ventricular end-diastolic diameter; LVEDS, left ventricular end-systolic diameter; LVEF, left ventricular ejection fraction; IVS, interventricular septum thickness; LVOTO, left ventricular outflow tract obstruction; LVOTG, left ventricular outflow tract gradient; LVPW, left ventricular posterior wall thickness; AADs, antiarrhythmic drugs; *, Fisher’s exact test; NA, not applicable.

### 3.2 Ablation Procedure

The details of the ablation procedures are summarized in Table 2. CPVI was achieved in all patients. Substrate modification-encompassing linear ablation and/or fractionated potential ablation-was performed more frequently in the aHCM cohort (30.6% vs.15.0%, *p* = 0.052). Cavotricuspid isthmus isolation and superior vena cava isolation were similar between groups (8.3% vs. 17.5%; 5.6% vs. 6.3%, both *p* > 0.05).

**Table 2.** **Procedure parameters and ATa recurrence**.

Variables	All patients	sHCM	aHCM	*p*-value
	(n = 116)	(n = 80)	(n = 36)	
PV isolation, n (%)	116 (100)	80 (100)	36 (100)	NA
Substrate modification, n (%)	23 (19.8)	12 (15.0)	11 (30.6)	0.052
CTI isolation, n (%)	17 (14.7)	14 (17.5)	3 (8.3)	0.197
SVC isolation, n (%)	7 (6.0)	5 (6.3)	2 (5.6)	0.884
Complications, n (%)	1 (0.8)	1 (1.3)	0 (0.0)	1.000*
Follow-up months	42 (18, 83)	43 (23, 85)	38 (13, 78)	0.172
ATa recurrence, n (%)	49 (42.2)	28 (35.0)	21 (58.3)	0.019

PV, pulmonary vein; CTI, cavotricuspid isthmus; SVC, superior vena cava; Ata, atrial tachycardias; *, Fisher’s exact test; NA, not applicable.

### 3.3 Follow-up

**Fig. 2.**
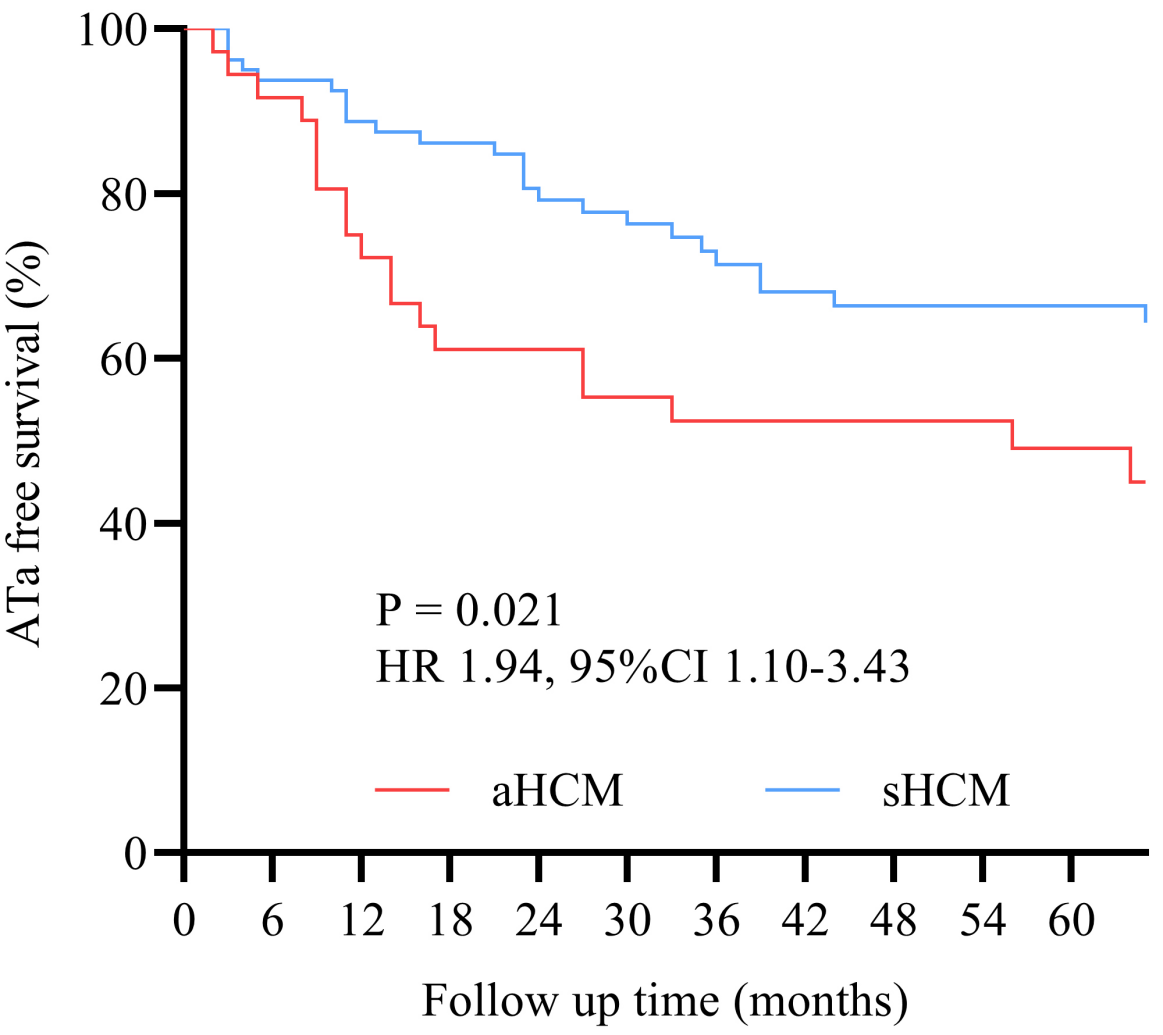
**Kaplan–Meier survival curves**. Kaplan–Meier analysis revealed a 94% increased cumulative ATa recurrence risk in the aHCM group compared to the sHCM group (HR 1.94, 95% CI 1.10–3.43; log-rank *p* = 0.021).

**Fig. 3.**
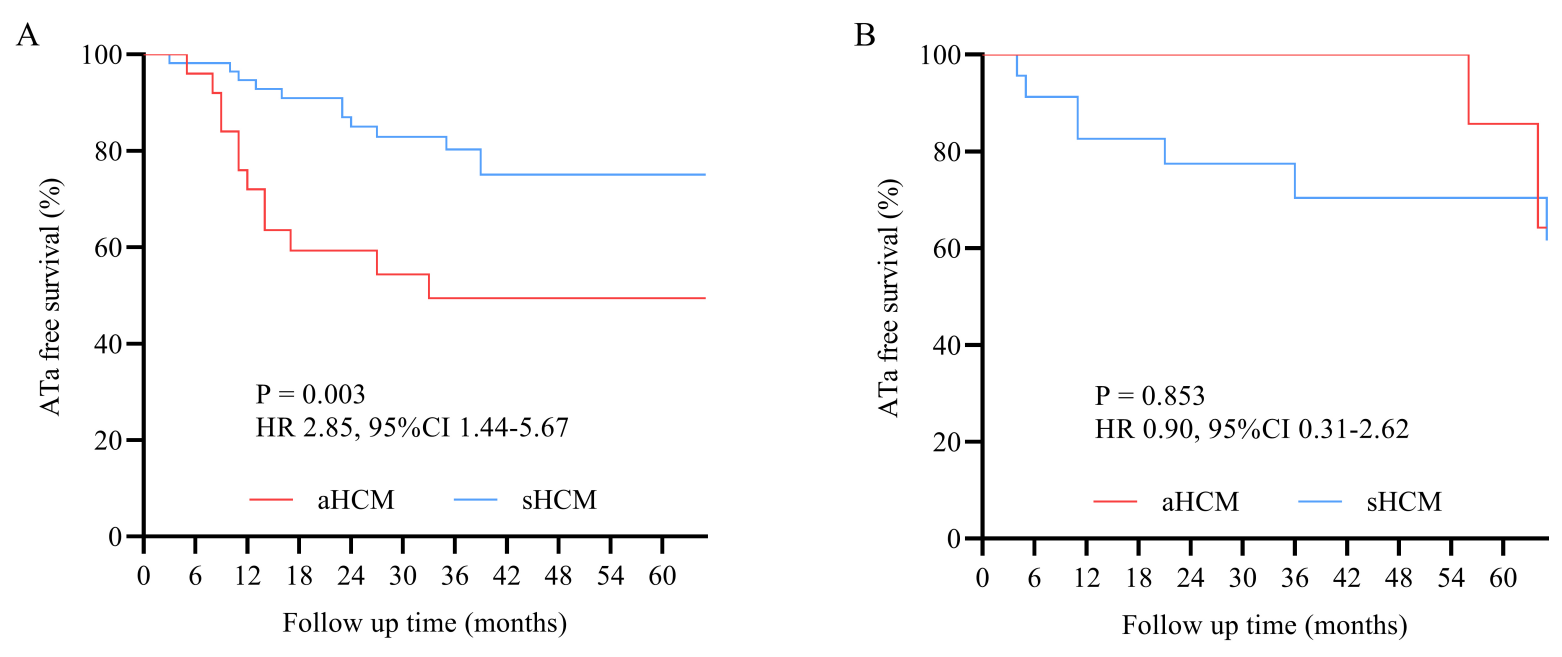
**Kaplan–Meier survival curves**. (A) sHCM demonstrated superior outcomes versus aHCM in paroxysmal AF (*p* = 0.003). (B) No significant intergroup difference observed in persistent AF (*p* = 0.853).

**Fig. 4.**
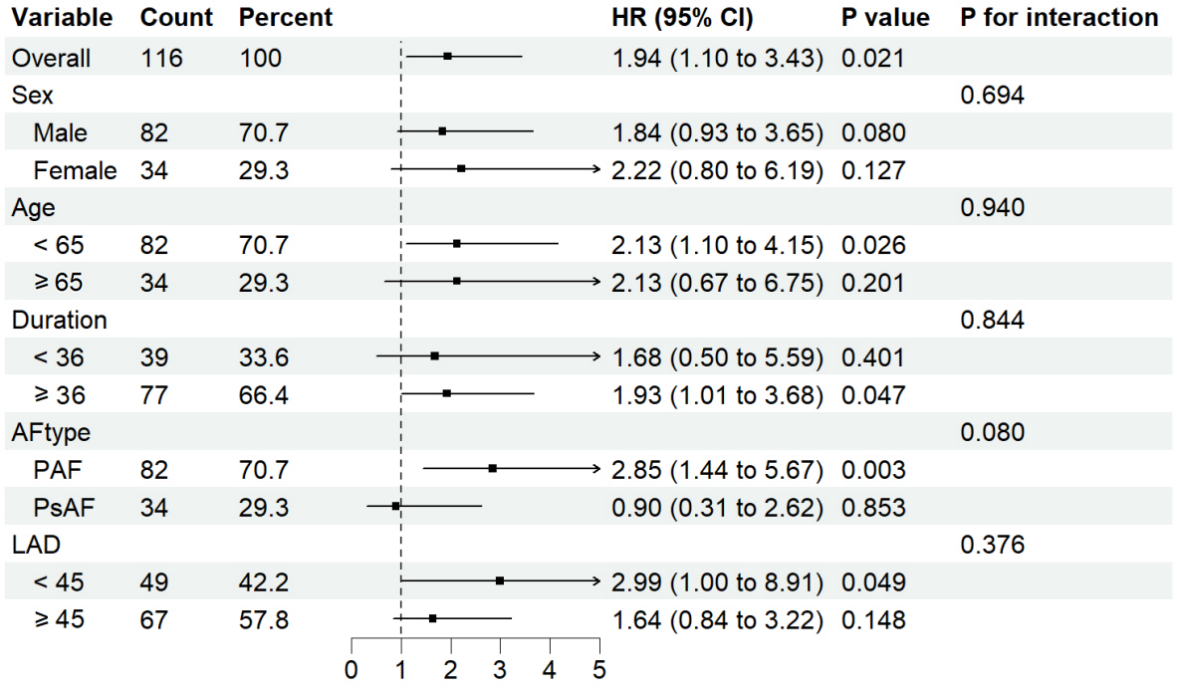
**Subgroup heterogeneity analysis**. The forest plot demonstrates a non-significant interaction effects across prespecified subgroups (*p* for interaction > 0.05).

Overall ATa recurrence rate was 42.2% (49/116), after a median follow-up of 42 (18, 83) months. The aHCM group demonstrated significantly higher ATa recurrence rates than the sHCM group [58.3% (21/36) vs. 35.0% (28/80); χ^2^ = 5.54; *p* = 0.019]. Kaplan–Meier analysis revealed a 94% increased cumulative recurrence risk in the aHCM group compared to the sHCM group (HR 1.94, 95% CI 1.10–3.43; log-rank *p* = 0.021) (Fig. 2). Subgroup analysis showed this disparity was particularly pronounced in paroxysmal AF patients (HR 2.85, 95% CI 1.44–5.67; *p* = 0.003) (Fig. 3A), while no significant intergroup difference was observed in persistent AF patients (HR 0.90, 95% CI 0.31–2.62; *p* = 0.853) (Fig. 3B). Subgroup interaction analysis revealed no statistically significant heterogeneity (interaction *p* = 0.080) (Fig. 4).

### 3.4 Predictors of Recurrence

Patients with ATa recurrence exhibited longer AF duration, higher prevalence of hypertension, larger left atrial diameter (≥45 mm), increased left ventricular end-diastolic dimension (LVEDD ≥47 mm), higher rates of substrate modification and aHCM, along with lower AAD utilization (Table 3). Variables meeting the threshold of univariate *p* < 0.1 and clinical relevance were incorporated into multivariate Cox regression analysis. The results identified four independent predictors of postoperative recurrence: AAD usage (HR 0.22, 95% CI 0.08–0.59; *p* = 0.003), comorbid hypertension (HR 2.50, 95% CI 1.21–5.19; *p* = 0.014), AF type (HR 0.41, 95% CI 0.17–1.00; *p* = 0.049), and left atrial enlargement (≥45 mm) (HR 2.55, 95% CI 1.11–5.85; *p* = 0.028) (Table 4).

**Table 3.** **Patient characteristics according to ATa recurrence following initial catheter ablation**.

Variables	With recurrence	Without recurrence	*p*-value
	(n = 35)	(n = 81)	
Age (yrs)	60.2 ± 8.3	56.9 ± 12.2	0.096
Male, n (%)	26 (74.3)	56 (69.1)	0.576
Height (cm)	171.0 ± 7.5	170.4 ± 8.0	0.695
Weight (kg)	71.4 ± 11.0	70.3 ± 10.5	0.595
Persistent AF, n (%)	10 (28.6)	24 (29.6)	0.909
AF duration (months)	67.1 ± 49.6	53.4 ± 71.9	0.309
Hypertension, n (%)	23 (65.7)	31 (38.3)	0.007
Diabetes, n (%)	6 (17.1)	15 (18.5)	0.860
LAD ≥45 mm, n (%)	27 (77.1)	40 (49.4)	0.005
RAD (mm)	36.2 ± 4.2	37.2 ± 4.8	0.301
LVEDD ≥47 mm, n (%)	26 (74.3)	47 (58.0)	0.096
LVEDS (mm)	30.6 ± 5.5	30.6 ± 3.7	0.990
IVS (mm)	16.4 ± 3.5	16.0 ± 3.6	0.566
LVEF (%)	63.5 ± 3.5	63.5 ± 4.4	0.928
LVOTO (%)	7 (8.6)	2 (5.7)	0.588
AADs (n)	29 (82.9)	80 (98.8)	0.001
β receptor blocker, n (%)	25 (71.4)	65 (80.2)	0.296
ACEI/ARB, n (%)	11 (31.4)	29 (35.8)	0.649
Calcium antagonist, n (%)	6 (17.1)	11 (13.6)	0.618
NOACs, n (%)	12 (34.3)	27 (33.3)	0.921
Warfarin, n (%)	23 (65.7)	46 (56.8)	0.369
aHCM, n (%)	15 (42.9)	21 (25.9)	0.070
Substrate modification, n (%)	11 (31.4)	12 (14.8)	0.039
CTI isolation, n (%)	3 (8.6)	14 (17.3)	0.223
SVC isolation, n (%)	0	7 (8.6)	0.173

AF, atrial fibrillation; ACEI/ARB, angiotensin-converting enzyme inhibitor/angiotensin II receptor blocker; NOACs, non-vitamin K antagonist oral anticoagulants; LAD, left atrial diameter; RAD, right atrial diameter; LVEDD, left ventricular end-diastolic diameter; LVEDS, left ventricular end-systolic diameter; LVEF, left ventricular ejection fraction; LVOTO, left ventricular outflow tract obstruction; AADs, antiarrhythmic drugs; CTI, cavotricuspid isthmus; SVC, superior vena cava; sHCM, septal hypertrophic cardiomyopathy.

**Table 4.** **Predictors of ATa recurrence**.

Variables	HR	95% CI	*p*-value
AAD	0.22	0.08–0.59	**0.003**
Hypertension	2.50	1.21–5.19	**0.014**
LAD ≥45 mm	2.55	1.11–5.85	**0.028**
LVEDD ≥47 mm	0.84	0.36–1.97	0.690
HCM type (aHCM)	1.49	0.07–3.14	0.299
AF duration	1.00	0.99–1.01	0.284
AF type (PeAF)	0.41	0.17–1.00	**0.049**
Substrate modification	0.28	0.69–1.57	0.590
mitral regurgitation	1.55	0.57–4.21	0.395

AADs, antiarrhythmic drugs; AF, atrial fibrillation; LAD, left atrial diameter; LVEDD, left ventricular end-diastolic dimension; sHCM, septal hypertrophic cardiomyopathy; AF, atrial fibrillation. Bold values: *p* < 0.05.

## 4. Discussion

### 4.1 Main Findings

This study revealed three principal findings: (1) The overall ATa recurrence after a single procedure was 42.2% over a nearly four-year follow-up period. (2) aHCM patients had a significantly higher recurrence rate than those with sHCM, primarily driven by the presence of paroxysmal AF. (3) Hypertension, left atrial enlargement, and no AAD use were identified as independent predictors of recurrence.

### 4.2 Validation of Catheter Ablation Efficacy

Our overall success rate (57.8%) aligns with prior HCM ablation studies [6, 7, 15, 16, 17]. Notably, despite requiring multiple procedures, success rates in HCM patients remained substantially lower than in non-HCM populations [8], underscoring the impact of HCM-specific myocardial substrate on ablation outcomes. HCM-driven atrial remodeling occurs through two main pathways: first, structural changes such as left atrial wall hypertrophy and pulmonary venous sleeve dysplasia [16, 18]; and second, hemodynamic stress resulting from diastolic dysfunction, which leads to progressive left atrial dilation. Together, these mechanisms contribute to electromechanical remodeling, characterized by low-voltage zones and increased complex fractionated electrograms.

### 4.3 Resolving Controversies in HCM Subtype Prognosis

Evidence regarding the outcomes of AF ablation across various subtypes of HCM remains inconsistent and conflicting [19, 20]. Our data show that ATa recurrence is higher in patients with aHCM compared to those with sHCM. We propose three potential explanations for this observation. First, aHCM demonstrates a greater presence of low-voltage zones; our findings support this, showing that substrate modification occurred twice as frequently in aHCM patients (30.6% compared to 15.0% in sHCM). Second, electromechanical dysfunction associated with apical hypertrophy leads to impaired ventricular relaxation, which increases left atrial pressure and exacerbates atrial stretch and electrical instability. Third, the higher prevalence of hypertension in aHCM patients may further compound the risk of recurrence, with hypertension independently predicting recurrence (HR 2.50).

### 4.4 Pronounced Risk in Paroxysmal AF

The pronounced risk associated with paroxysmal AF, indicated by a hazard ratio of 2.85, contrasts with persistent AF, which showed no significant difference (*p* = 0.853), suggesting that triggers may be phenotype-specific. In paroxysmal AF, the condition primarily arises from the pulmonary veins, and the distinct fibrosis creates arrhythmogenic substrates that extend beyond the pulmonary veins, potentially harboring non-pulmonary vein triggers that are resistant to standard ablation techniques. In contrast, persistent AF is influenced by a more diffuse substrate, and both phenotypes exhibit advanced remodeling, which may dilute the differences observed between them. Clinically, this implies that patients with aHCM and paroxysmal AF might benefit from first-line extensive substrate ablation strategies, such as posterior wall isolation and voltage mapping. Our strict criteria for sHCM, focusing on reverse-curve septal hypertrophy, allow for a clearer isolation of phenotype effects.

### 4.5 Novel Risk Stratification Markers and Clinical Implications

While left atrial enlargement remains a key predictor of recurrence [21, 22, 23], our study has identified hypertension (adjusted Hazard Ratio (aHR) 2.50, 95% CI 1.21–5.19) and AAD usage (aHR 0.22, 95% CI 0.08–0.59) as independent risk modifiers. Hypertension may accelerate fibrosis via RAAS overactivation and autonomic dysregulation, while the maintenance of AAD usage likely stabilizes post-ablation substrates. Antihypertensive therapy could reduce both the incidence of *de novo* AF and post-cardioversion/ablation recurrence, particularly when targeting optimal blood pressure control [24]. As hypertension represents a modifiable risk factor, optimized blood pressure control (target <130/80 mmHg) and using RAAS inhibitors (supported by AF prevention trials [25, 26, 27, 28]) may enhance ablation success in HCM patients.

### 4.6 Evidence Gap & Current Approach

Performing concomitant AF surgery during septal myectomy effectively eliminates AF in patients with HOCM [29]. However, this study specifically excluded patients with more severe forms of HCM that would necessitate surgical intervention. The majority of our cohort consisted of patients with mild hypertrophy, with an average septal thickness of 16.1 ± 3.6 mm, many of whom exhibited latent obstruction. Due to the lack of dedicated comparative studies for these specific subgroups, and the established efficacy and safety of catheter ablation, catheter-based AF ablation currently stands out as the preferred approach for managing symptomatic AF in these patients. This recommendation aligns with the broader principle of prioritizing rhythm control strategies that address the underlying disease mechanisms while also minimizing procedural complexity.

### 4.7 Limitations

A significant limitation of this study is the absence of a systematic assessment of late gadolinium enhancement (LGE). CMRI-LGE is considered the gold standard for measuring fibrosis in both the atria and ventricles. This fibrosis has been linked to the complexity of the AF substrate and the ablation outcomes in HCM [30, 31]. Without LGE data, we could not evaluate whether the differences in fibrotic burden contribute to the varying efficacy observed between apical and septal phenotypes. Therefore, future research should incorporate LGE assessments to accurately identify patients who might benefit from additional substrate modification strategies. The study has several additional limitations: First, the single-center retrospective design increases the risk of selection bias and residual confounding, particularly concerning the phenotyping of HCM and the standardization of ablation strategies. Second, the moderate sample size of 116 participants limits the robustness of subgroup analyses. Third, genetic factors such as *MYBPC3* and *MYH7* mutations were not systematically assessed. Additionally, the absence of left atrial strain parameters and the reliance on left ventricular end-diastolic dimension measurements, rather than volumetric assessments may inadequately characterize diastolic dysfunction in HCM. Furthermore, 24-hour Holter monitoring may underestimate asymptomatic recurrences or intermittent ATa episodes. Lastly, the lack of quantitative symptom assessment restricts our ability to evaluate the clinical benefits of ablation beyond merely measuring arrhythmia recurrence. Previous studies have shown that AF ablation can enhance symptom alleviation even in cases of recurrent ATa [32]. Therefore, future research needs to incorporate patient-reported outcomes to provide a more comprehensive understanding of the treatment’s impact.

## 5. Conclusions

Our study indicates that patients with aHCM face a significantly greater risk of ATa recurrence after ablation compared to those with sHCM. This notable difference in recurrence rates for paroxysmal AF underscores the need to recognize the specific vulnerabilities associated with different phenotypes. Consequently, our findings suggest that treatment strategies should be tailored to individual phenotypes. Additionally, maintaining strict control of hypertension, aiming for a blood pressure of less than 130/80 mmHg, along with the use of AADs after the ablation, is essential. Future research should focus on validating the benefits of adjunctive ablation techniques and the use of CMRI to quantify fibrosis, as these approaches could significantly enhance outcomes for this high-risk patient group.

## Availability of Data and Materials

The data presented in this study are available on request from the corresponding author.
